# Geriatric assessment and intervention in older vulnerable patients undergoing surgery for colorectal cancer: a protocol for a randomised controlled trial (GEPOC trial)

**DOI:** 10.1186/s12877-021-02045-9

**Published:** 2021-01-30

**Authors:** Troels G. Dolin, Marta Mikkelsen, Henrik L. Jakobsen, Tyge Nordentoft, Trine S. Pedersen, Anders Vinther, Bo Zerahn, Kirsten K. Vistisen, Charlotte Suetta, Dorte Nielsen, Julia S. Johansen, Cecilia M. Lund

**Affiliations:** 1 Department of Medicine, Copenhagen University Hospital, Herlev and Gentofte, Borgmester Ib Juuls Vej 1, DK-2730 Herlev, Denmark; 2 CopenAge – Copenhagen Center for Clinical Age Research, University of Copenhagen, Copenhagen, Denmark; 3 Department of Oncology, Copenhagen University Hospital, Herlev and Gentofte, Copenhagen, Denmark; 4 Department of Surgery, Copenhagen University Hospital, Herlev and Gentofte, Copenhagen, Denmark; 5 Department of Physiotherapy and Occupational Therapy, Copenhagen University Hospital, Herlev and Gentofte, Copenhagen, Denmark; 6 Hospital Secretariat and Communications, Research, Copenhagen University Hospital, Herlev and Gentofte, Copenhagen, Denmark; 7 Department of Clinical Physiology and Nuclear Medicine, Copenhagen University Hospital, Herlev and Gentofte, Copenhagen, Denmark; 8 Department of Geriatric and Palliative Medicine, Copenhagen University Hospital, Bispebjerg and Frederiksberg Hospital, Copenhagen, Denmark; 9 Department of Clinical Medicine Faculty of Health and Medical Sciences, University of Copenhagen, Copenhagen, Denmark

**Keywords:** Colorectal cancer, Comprehensive geriatric assessment, Surgery, Frailty, Sarcopenia, Exercise

## Abstract

**Background:**

The incidence of colorectal cancer (CRC) increases with age. Older patients are a heterogeneous group ranging from fit to frail with various comorbidities. Frail older patients with CRC are at increased risk of negative outcomes and functional decline after cancer surgery compared to younger and fit older patients. Maintenance of independence after treatment is rarely investigated in clinical trials despite older patients value it as high as survival. Comprehensive geriatric assessment (CGA) is an evaluation of an older persons’ medical, psychosocial, and functional capabilities to develop an overall plan for treatment and follow-up. The beneficial effect of CGA is well documented in the fields of medicine and orthopaedic surgery, but evidence is lacking in cancer surgery. We aim to investigate the effect of CGA on physical performance in older frail patients undergoing surgery for CRC.

**Methods:**

GEPOC is a single centre randomised controlled trial including older patients (≥65 years) undergoing surgical resection for primary CRC. Frail patients (≤14/17 points using the G8 screening tool) will be randomised 1:1 to geriatric intervention and exercise (*n* = 50) or standard of care along (*n* = 50) with their standard surgical procedure. Intervention includes preoperative CGA, perioperative geriatric in-ward review and postoperative follow-up. All patients in the intervention group will participate in a pre- and postoperative resistance exercise programme (twice/week, 2 + 12 weeks). Primary endpoint is change in 30-s chair stand test. Assessment of primary endpoint will be performed by physiotherapists blinded to patient allocation. Secondary endpoints: changes in health related quality of life, physical strength and capacity (handgrip strength, gait speed and 6 min walking test), patient perceived quality of recovery, complications to surgery, body composition (Dual-energy X-ray absorptiometry and bioelectric impedance), serum biomarkers, readmission, length of stay and survival.

**Discussion:**

This ongoing trial will provide valuable knowledge on whether preoperative CGA and postoperative geriatric follow-up and intervention including an exercise program can counteract physical decline and improve quality of life in frail CRC patients undergoing surgery.

**Trial registration:**

Prospectively registered at Clinicaltrials.gov NCT03719573 (October 2018).

**Supplementary Information:**

The online version contains supplementary material available at 10.1186/s12877-021-02045-9.

## Background

Colorectal cancer (CRC) is the third most common cancer worldwide accounting for 1.8 million new cases in 2018 [1]. The incidence of CRC increases with age [2], with a median age at time of diagnosis of 70 years in Denmark [3]. As the population is getting older, an increasing number of older patients with CRC is expected in the future [4, 5].

The mainstay in treatment of localised CRC is resection [6]. Implementation of better perioperative care, minimal invasive surgery and enhanced recovery pathways have made cancer surgery available for a larger proportion of patients with CRC [7]. Surgery itself and complications from surgery increase the risk of functional decline and loss of independence which can be difficult to regain [8]. The burden of treatment as well as possible treatment outcomes are strong determinants of older patients’ preferences [9]. Maintenance of independence and health related quality of life represent preferred treatment outcomes over standard oncological outcomes (e.g. disease free survival) for older patients [9–11]. In general, surgery is considered safe for older patients and the primary risk factor for poor surgical outcome, usually defined as surgical complications or survival, is not age but comorbidity [12], frailty [13, 14] and sarcopenia [15]. However, loss of independence is reported in 1 in 5 patients older than 65 years undergoing surgery for CRC [8, 16].

Frailty is recognised as an independent risk factor and has been described as “A medical syndrome with multiple causes and contributors that is characterized by diminished strength, endurance, and reduced physiologic function that increases an individual’s vulnerability for developing increased dependency and/or death” [17]. Frail patients are more susceptible to adverse effects from external stressors such as surgery [13]. Identifying frail people is therefore crucial for more careful planning of surgery and treatment. Merely screening for frailty brings awareness of patient comorbidity and capacity and is associated with reduced mortality after surgery [18]. Frailty is not a permanent state but potentially reversible [19] since some comorbidities are modifiable, as are both physical capacity and nutritional status.

Comprehensive Geriatric assessment (CGA) is hitherto the most appropriate way to examine the overall health situation of the older frail patient [20]. CGA is a multidomain and usually multidisciplinary work tool used for systematic assessment of an older person’s medical, psychosocial, as well as functional capabilities and limitations in order to develop an overall plan for interventions and follow-up [21]. CGA assesses known pathology and can uncover unrecognized health issues [22]. Furthermore, a prospective study has shown that CGA can assess the risk of postoperative complications in older patients undergoing elective CRC surgery [23]. Nonetheless, CGA is time consuming and may not be needed for all older patients. Several more rapid frailty screening tools have therefore been developed to identify which patients will benefit from a CGA [24]. The recommendations from the International Society for Geriatric Oncology (SIOG) from June 2014 [25] find the G8 questionnaire to be equally sensitive or superior when compared to other screening tools. For patients with an abnormal G8 score (≤ 14/17) a full CGA is recommended [26, 27].

One of the most important factors leading to frailty is low muscle strength, functional performance and low muscle mass (i.e. sarcopenia) [17]. Progressive resistance training is the single most effective intervention to increase muscle strength, muscle mass and functional performance in healthy older individuals [28] and in different patient populations [29–32]. Physical exercise is therefore recommended by WHO [33] and is shown to be safe and beneficial among older patients with cancer [34, 35].

Yet, there is a need for randomised controlled trials investigating the effect of the CGA in cancer surgery with patient-centred outcomes as preferred by the older patients. Thus, the aim of the present study is to evaluate the effect of pre-operative CGA and physical exercise, post-operative geriatric in-ward review and exercise, and post-operative geriatric rehabilitation on patients´ physical and functional performance.

## Methods/design

### Trial design

**Fig. 1:**
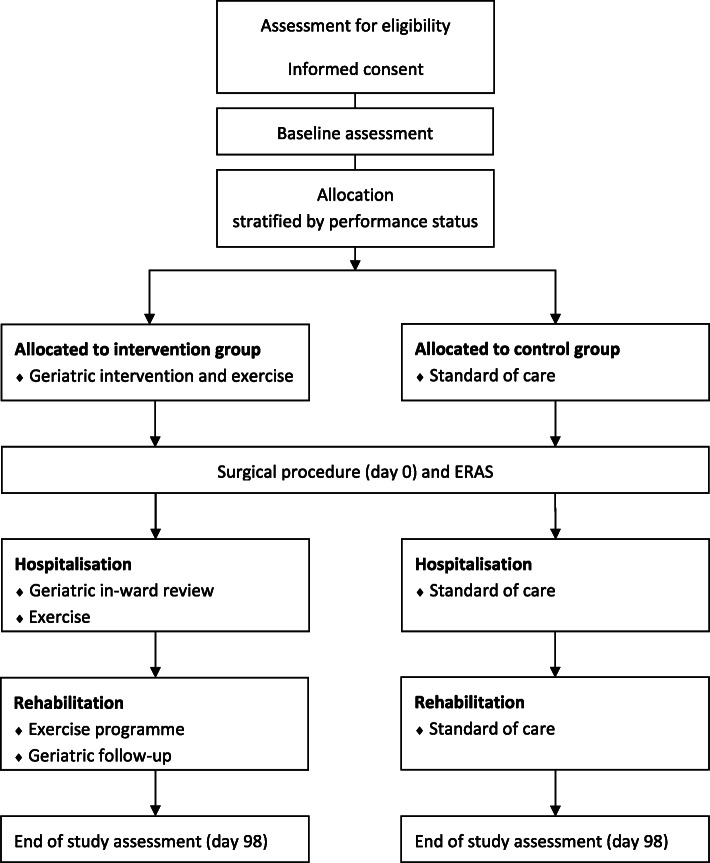
GEPOC trial design. Abbreviation: ERAS Enhanced Recovery After Surgery

This is a randomised controlled trial (RCT) comparing a multidisciplinary intervention to standard of care in older frail patients with CRC undergoing curative intended surgery. The trial design follows the SPIRIT guidelines for randomised controlled trials. Trial design can be seen in Fig. 1.

### Participants

A total of 100 patients will be recruited from a single tertiary surgical centre at Herlev and Gentofte Hospital, Copenhagen University Hospital.

#### Inclusion criteria

For inclusion in the study, all the following must be fulfilled: (i) age ≥ 65 years; (ii) frailty evaluated with a G8 questionnaire score ≤ 14/17; (iii) scheduled for elective primary CRC surgical resection; and (iv) histologically proven adenocarcinoma.

#### Exclusion criteria

None of the following should apply if the patient is eligible for the study: (i) neoadjuvant treatment; (ii) severe physical disability that hinders training; and (iii) inability to give informed consent.

### Randomisation

Randomisation is performed electronically in REDcap randomisation module from permuted blocks and stratified for Eastern Cooperative Oncology Group (ECOG) performance status. Patients will be randomized 1:1 to an intervention group and a control group.

### Management in both groups

All patients will be evaluated at the multidisciplinary team (MDT) conference before surgery. Surgical resection is according to national guidelines [6] and follow the local Enhanced Recovery After Surgery (ERAS) programme with minimal invasive surgery [36]. The ERAS programme also includes pre-operative participation in the ERAS school where patients receive (i) education from a nurse specialist in preparation for surgery, (ii) education about nutrition from a dietician and (iii) education on mobilisation and lung physiotherapy from a physiotherapist. After surgery the ERAS programme includes early mobilisation and oral nutrition at the surgical ward. The geriatric intervention will not affect the surgical planning (e.g. laparoscopic or open surgery) nor the timing for surgery.

### Geriatric intervention

All patients in the intervention group will receive a three-phased intervention: pre-operative-, peri-operative- and post-operative. All phases consist of geriatric interventions including a physical exercise programme.

#### Geriatric assessment and tailored interventions

The pre-operative phase consists of a home visit with a CGA with tailored interventions on identified health issues. The contents of the geriatric assessment is based on recommendations from SIOG [37]. The following domains are assessed: comorbidity, medication, social status, cognition, psychological status, functional status and nutrition. Assessment tools and possible interventions can be seen in Supplement 1.

The peri-operative phase includes post-operative geriatric review in addition to surgical review in the surgical ward. This includes early detection and treatment of medical complications (e.g. delirium, infections, atrial fibrillation) and multidisciplinary issues (e.g. discharge planning, pain management, nutrition).

The post-operative phase: Geriatric follow-up on identified health problems if needed.

#### Exercise programme

All phases of the programme are supervised by experienced physiotherapists.

Pre- and post-operative phases: supervised group-based exercise programme scheduled twice a week from inclusion until surgery. After a two-week post-operative pause the programme continues for further 12 consecutive weeks. The exercise programme is a 1-h programme including 15 min warming up, 35 min of progressive resistance training followed by 10 min of cool down. The progressive resistance training focuses on large muscle groups and follows recommendations from American College of Sports Medicine [38–40]. The programme has previously been described by Mikkelsen et al. [41] The pre- and post-operative phases of exercise are both conducted in the Department of Physiotherapy and Occupational Therapy, Herlev Hospital.

Perioperative phase: patients will receive supervised training from physiotherapists throughout the stay when admitting on the surgical ward. The training programme is adapted to each individual in accordance with the surgical procedure (e.g. laparoscopic or laparotomic approach) and conferred with the surgical staff before each session. The exercise programme is conducted in the surgical ward. Details can be seen in Supplement 2. After discharge patients will be provided with an activity tracker. They will be guided in setting goals in relation to activity throughout the study.

### Endpoints and measurements

**Fig. 2:**
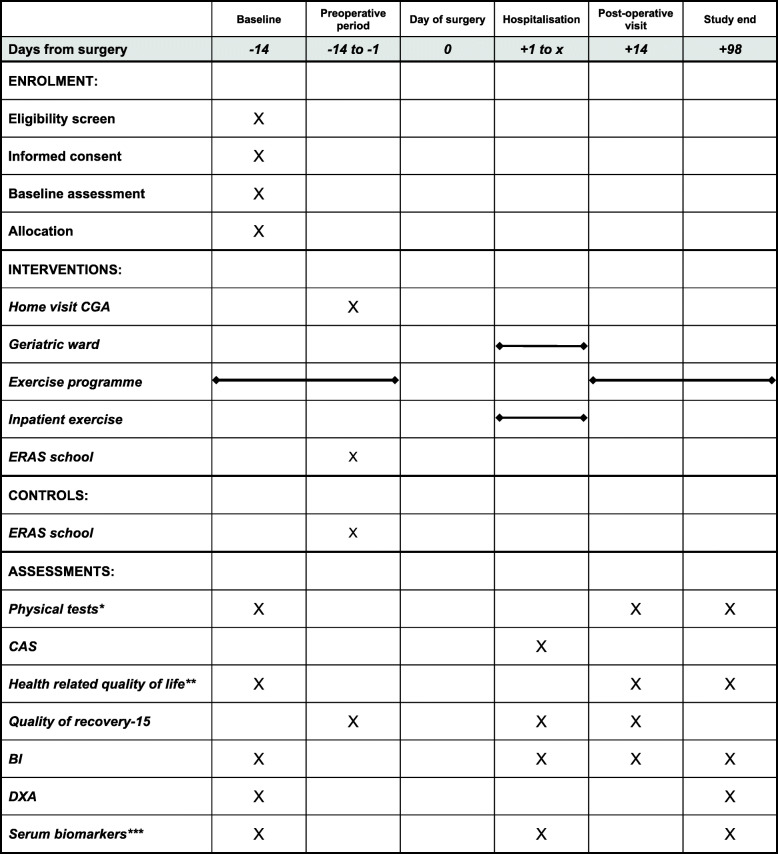
SPIRIT flow diagram. Abbreviations: CGA Comprehensive Geriatric Assessment, ERAS Enhanced Recovery After Surgery, CAS Cumulated Ambulation Score, BI Bioelectric Impedance, DXA Whole-body dual-energy X-ray absorptiometry. * Physical tests: 30-s chair stand test, 6 + 10-m usual gait speed, handgrip strength, 5-times-sit-to-stand, six-minute-walk-test. ** questionnaires: European Organization for Research and Treatment of Cancer Quality of Life Questionnaire Core 30 (EORTC QLQ-C30) + Elderly Cancer Patients Module (EORTC QLQ-ELD14). *** Serum biomarkers: Interleukin-6, YKL-40, C-reactive Protein, Olink panel, Growth Differentiation Factor 11 + 14, Vitamin D

Baseline test prior to randomisation includes test of physical strength and capacity, questionnaire of quality of life and estimation of body composition. Study data are collected on the 2. and 14. postoperative day, and at the end of study 14 weeks after surgery (Fig. 2).

### Primary endpoint

The primary endpoint of the GEPOC study is change in 30-s Chair Stand Test (30s-CST) from baseline to 14 weeks after surgery. The 30s-CST measures a combination of muscle strength and endurance and is highly sensitive in older patients [42]. A high test-score is regarded as a predictor for functional independence in older patients [43–45] as it measures the ability to rise from a chair. The 30s-CST has also been used and tested in cancer patients [46, 47].

### Secondary endpoints

1. **Muscle strength and functional capacity**: changes in hand grip strength, 6 m and 10 m habitual gait speed, 5-times-sit-to-stand and 6-min-walk-test. The 5-times-sit-to-stand is performed separately in accordance with latest guidelines for sarcopenia [48]**.** Changes in the physical tests will be reported from baseline at two and 14 weeks after surgery and changes from 2 weeks after surgery at 14 weeks after surgery. Additional information regarding the tests are described in Supplement 3. Cumulated Ambulation Score (CAS) [49] will be measured in the postoperative period, as a measurement of day-to-day basic mobility. For acute abdominal surgery a poor CAS is associated with pulmonary complications and low activity in the first postoperative week [50] and CAS has been shown to be predictive for discharge to a higher level of dependence [49]. CAS will be compared between groups and investigated as a predictor for poor postoperative outcome independent of patient allocation to control or intervention group. All physical tests will be performed in the same sequence by a physiotherapist blinded to patients’ allocation to control or intervention group.
2. **Patient reported outcome measures:** Patient’s perspective of recovery in the perioperative period will be assessed with Quality of Recovery-15 (QoR-15). QoR-15 is a patient reported outcome questionnaire that quantifies recovery from surgery and anaesthesia [51, 52].
  Health related Quality of Life (HRQoL) will be assessed with European Organization for Research and Treatment of Cancer Quality of Life Questionnaire Core 30 (EORTC QLQ-C30) conjoined with the module for older patients with cancer (QLQ-ELD14). The questionnaires incorporate functional scales, symptom scales and a global health and quality-of-life scale [53, 54].
3. **Postoperative complications (30 + 90 days):** The occurrence of postoperative complication within 30 + 90 days after surgical resection of CRC will be graded from 0 to 5 based on the classification system validated by Clavien and Dindo [55]. Further details are described in Supplement 4.
4. **Body composition and sarcopenia:** Whole-body dual-energy X-ray absorptiometry (DXA) scan (GE lunar iDXA, GE Healthcare Technologies, Madison, Wisconsin, U.S.) and bioelectrical impedance (BI) assessment (Body Composition Monitor, Fresenius Medical Care, Bad Homburg v.d.H., Germany) will be used to measure body composition. Sarcopenia will be reported according to the revised guidelines from the European Working Group on Sarcopenia in Older People [48].
5. **Length of stay and readmission rate:** The postoperative length of stay defined from the day of surgical resection of CRC to the day of discharge from the surgical ward will be calculated. Readmission rate is defined as any unplanned hospitalisation within 30 and 90 days of discharge is registered.
6. **Initiation and completion of adjuvant chemotherapy:** Patients will be treated with adjuvant chemotherapy according to national guidelines if indication for chemotherapy is found [56]. Type of chemotherapy, dose, number of series and adverse events are registered.
7. **Survival and mortality:** Cancer- and treatment-related mortality or other cause of mortality will be registered throughout the study and a during a follow-up period of 5 years.
8. **Serum biomarkers:** Blood samples will be taken 1–3 days before surgery, at the first and second postoperative day and at the end of study. Serum biomarkers will be analysed as part of cooperative research project REBECCA (“Biomarkers for patients with colorectal cancer - providing new information on diagnosis, treatment effect, side-effects and prognosis” (translated from Danish)). The REBECCA study is approved by the Regional Ethics Committee (H-2-2013-078) and the Danish Data Protection Agency (jr. nr. 2007-58-0015; HEH-2014-044; I-suite nr. 02771 and PACTIUS P-2019-614).

To investigate the influence of CGA and CGA-based exercise for older frail patients with CRC the following serum biomarkers will be measured: C-reactive protein (CRP), interleukin-6 (IL-6), YKL-40, Growth Differentiation Factor (GDF) 11 and 15, vitamin D and the Olink immune-oncology panel [57] (a panel of 92 serum proteins proteins associated with inflammation and immunology and cancer). Further details are given in Supplement 5.

### Statistical analysis

#### Sample size

Calculation of statistical power is based on the estimated effect of CGA based interventions on physical performance. According to a previous study focusing on patients with osteoarthritis, the clinically relevant change in the 30s-CST was set at 2.6 repetitions [58]. Based on results from prior studies focusing on patients with advanced cancer, a standard deviation (SD) of around 3 has been reported in the 30s-CST [59, 60]. To be able to detect a difference of 2.6 repetitions in the between-group difference in the 30s-CST at the 12-week assessment and to obtain a type I error rate of 5% and a power of 90%, a sample size of 29 patients per study arm will be needed. To account for an expected dropout rate of ~ 40%, we decided to increase this number to a group size of 50. Thus, the aim will be to include a total of 100 patients in the study.

#### Quantitative data

Results from physical tests, body composition measures, questionnaires and serum biomarkers will be reported as means and standard deviations (SD) or as median and IQR, as appropriate. Change over time in ordinal categorical values will be evaluated by a trend test using logistic regression. In-group and between- group differences in continuous-level data, will be performed using independent T-tests or the nonparametric Mann-Whitney U and Wilcoxon tests, depending on their distribution. Survival analyses will be conducted using Kaplan-Meier method, competing risk analyses and Cox regression analyses. Using the Kaplan-Meier method the cancer-specific mortality and overall survival (OS) will be assessed for the intervention group and the control group, and comparison in survival between groups will be assessed using the log-rank test.

Statistical analyzes will be performed by a statistician in collaboration with the primary investigator (TGD) using R Version 1.0.153.

### Trial status

The trial started in February 2019. Per primary January 2021 124 patients have been screened for frailty, 59 have been frail according to G8. 36 patients have been included. Amendments to the protocol have been made due to a lower rate of frail patients and a lower recruitment rate than anticipated. Age for inclusion have been lowered from 70 to 65 years (July 2019) and eligibility of patients with rectum cancer have been included (November 2019).

## Discussion

The present study is a RCT evaluating the effect of geriatric intervention in frail older patients undergoing surgery for primary CRC. The development of ERAS pathways and minimal invasive surgical technique have made surgery feasible for a broader population, including older patients. However, treatment decisions made today are often based on studies with younger and fitter patients, as the older frail population is often underrepresented in clinical trials [61]. Apart from observational studies [7], there is an urgent need for interventional studies focusing on patient-relevant outcomes as treatment goals for the older frail patients differ from their younger counterparts with an increased emphasis on independence, quality of life and physical capacity [9].

The CGA is a well-known tool in the intervention of older frail patients. In a meta-analysis of randomised trials, CGA-based interventions in patients admitted to the hospital for medical conditions increased the likelihood of being alive and living at home 6 months after hospital discharge [20]. The positive effect was greatest for geriatric wards, but geriatric teams also had a positive effect.

In orthopaedic surgery a beneficial effect in terms of 4 and 12  month physical function and increased independence was found for older patients with hip fractures randomised to comprehensive geriatric care compared to usual orthopaedic care [62]. For patients undergoing elective surgery, the incorporation of pre-operative CGA and post-operative inpatient geriatric review is seen in the UK with the Perioperative care of Older people undergoing Surgery (POPS) service. The POPS model was evaluated in a pre- and post-study and showed fewer postoperative medical complications, fewer multidisciplinary issues and a reduced length of hospitalisation [63]. The POPS concept was subsequently proved effective in a RCT of patients undergoing elective aorta or lower limb vascular surgery [64]. The RCT showed significantly reduced length of stay in the CGA group compared to standard care. The study did not use post-operative geriatric inpatient review but created a postoperative care plan providing advice on prevention and management of postoperative complications.

Few studies have investigated the effect of CGA in cancer surgery. In a recent Norwegian RCT, Ommundsen et al. [65] investigated the effect of preoperative geriatric intervention on postoperative complications (grade II-IV Clavien-Dindo classification) in patients undergoing elective surgery for CRC. No immediate difference was found between groups. When adjusted for prespecified prognostic factors, there was a statistically significant difference in favour of the intervention group when all complications (grade I-V) were taken into account. The study did not use post-operative geriatric inpatient review but made the geriatric assessment available to the surgical team and made general recommendations regarding medical and multidisciplinary issues (e.g. delirium and mobilisation). The study did not investigate the effect of CGA on functional status, quality of life or independence.

Older frail patients are prone to medical complications and loss of independence after surgery even if provided with optimal pre-operative care. Therefore, a major strength of the GEPOC study is expected to be the planned post-operative geriatric inpatient review. Furthermore, the broad collection of outcomes both in terms of physical testing, patient reported outcomes, body composition and serum biomarkers allow for a detailed insight in the effect of CGA based intervention in the population. To our knowledge no RCTs have been published investigating the effect of both pre-operative CGA and post-operative geriatric inpatient review for patients undergoing cancer surgery.

The CGA-based exercise programme is delivered throughout the GEPOC study. The pre-operative part is in line with the increasing interest in prehabilitation in the field of perioperative medicine. Prehabilitation is described by Carli et al. as “the process of enabling patients to withstand the stress of surgery through augmenting functional capacity” [66]. Prehabilitation focuses on preconditioning through exercise, nutrition, smoking cessation and anxiety reducing elements. A meta-analysis has shown effect on lean body mass [67], but results are still lacking in important outcomes e.g. complications to surgery, HRQOL, independence and physical capacity. Carli et al. showed no difference between groups in a RCT published in 2020 comparing prehabilitation to rehabilitation [68] but larger international studies are ongoing [69]. In the GEPOC study, the preconditioning period is short due to Danish cancer care plans which ensure surgery within 2 weeks from diagnosis. This challenges the ability to detect a measurable effect of the pre-operative exercise programme. However, the pre-operative physical exercise serves to improve neuromuscular adaptation and enable patients to get familiar with the exercise facilities and the training programme, thus facilitating a safe and effective return in the rehabilitation period. As older frail patients often have complex pathology going into surgery, a strength of CGA-based exercise in the GEPOC study is the assessment and individually tailored interventions ensuring a more personalised and holistic treatment approach also targeting possible modifiable barriers for exercise (e.g. dizziness as a result of too aggressive antihypertensive treatment). Furthermore, the effect of rehabilitation is well proven, and we hypothesize that combining prehabilitation and rehabilitation rather than only applying one of these would serve as a more beneficial treatment option for frail older patients. A limitation to this study setup is that it is unable to evaluate the effect of either prehabilitation or rehabilitation as a separate intervention.

Sarcopenia is reported in the GEPOC study and is regarded as one of the physical drivers of frailty. It is acknowledged as a separate entity characterised by muscle failure [70]. This is primarily defined by low muscle strength and can be confirmed by reduced muscle quantity and/or quality. If muscle functioning is impaired as well, sarcopenia is considered severe [48]. Sarcopenia has multiple contributing factors including the ageing process itself, insufficient nutrition, inactivity (sedentary lifestyle) and chronic diseases like cancer. The association between frailty and sarcopenia has not been fully elucidated, but they are linked with many of the same clinical outcomes e.g. low survival and complications to surgery [71]. It has also been suggested that the role of an aged immune system, with the age-related decline in immune function and a state of chronic inflammation, could play at potential role in the pathophysiology of both frailty and sarcopenia [72]. Interventions for treatment of sarcopenia are often multimodal with resistance exercise and diet advice on adequate protein and calorie intake [73, 74] which also are domains addressed in the CGA. In the GEPOC study we perform serial assessments of sarcopenia throughout the study period allowing for obtaining valuable information on the effect of CGA on sarcopenia. Identifying and treating sarcopenia could be a key factor in preventing negative implications of frailty in an ageing population of patients with cancer.

## Supplementary Information

**Additional file 1 Supplementary file 1.** Contents of the comprehensive geriatric assessment before surgery. **Supplementary file 2.** Exercise programme during hospitalisation. **Supplementary file 3.** Endpoints - Physical function tests. **Supplementary file 4.** Clavien and Dindo classification system for postoperative complication. **Supplementary file 5.** Serum biomarkers.

## Data Availability

Not Applicable.

## Abbreviations

ADL: Activities of daily living
BMI: Body mass index
BI: Bioelectrical impedance
CAM: Confusion Assessment Method
CAS: Cumulated Ambulation Score
CGA: Comprehensive Geriatric Assessment
CRP: C-reactive protein
CRC: Colorectal cancer
DXA: Dual-energy X-ray absorptiometry scan
EORTC: European Organisation for Research and Treatment of Cancer
EORTC QLQ-C30: European Organisation for Research and Treatment of Cancer Quality of Life Questionnaire- C30
GDF 11: Growth Differentiation Factor 11
GDF 15: Growth Differentiation Factor 15
GI: Geriatric intervention
HST: Handgrip strength test
IL-6: Interleukin 6
IQR: Interquartile range
MMSE: Mini Mental State Examination
PA: Physical activity
PROM: Patient-reported outcome measure
PRT: Progressive resistance training
PS: Performance status
RM: Repetition maximum
SD: Standard Deviation
SIOG: International Society of Geriatric Oncology
START: Screening Tool to Alert doctors to Right Treatment
STOPP: Screening Tool Older Person’s Prescriptions
QOL: Quality of life
30s-CST: 30-s chair stand test
6MGS: 6-m gait speed
6MWT: Six-minute-walk-test
