# Supplementary material for: Geriatric assessment and intervention in older vulnerable patients undergoing surgery for colorectal cancer: a protocol for a randomised controlled trial (GEPOC trial)
Source: BMC Geriatr. 2021 Jan 30;21:88. doi: 10.1186/s12877-021-02045-9 (PMC7847583; doi:10.1186/s12877-021-02045-9)
Supplement: Supplementary file 1 — Additional file 1 Supplementary file 1. Contents of the comprehensive geriatric assessment before surgery. Supplementary file 2. Exercise programme during hospitalisation. Supplementary file 3. Endpoints - Physical function tests. Supplementary file 4. Clavien and Dindo classification system for postoperative complication. Supplementary file 5. Serum biomarkers. [file 12877_2021_2045_MOESM1_ESM.docx]

Supplements (1-5) for manuscript:

**Geriatric assessment and intervention in older vulnerable patients undergoing surgery for colorectal cancer: a protocol for a randomised controlled trial (GEPOC trial)**

**Supplement 1:**

| **Tabel 1.** Comprehensive Geriatric Assessment before surgery | | |
| --- | --- | --- |
| **Domain** | **Tool/Assessment** | **Possible interventions** |
| Comorbidity | CIRS-G Medical record review  Physical examination  Biochemistry* | Change in treatment plans  Referral for further examination  Prioritising in hospital visits  Correction of iron deficiency in anaemia |
| Medication | START/STOPP Criteria | Discontinuation  Change in dosage  Assistance in medicine administration |
| Social status and demographic data | Home visit with assessment of resources and questions on living situation, social and family support | Further contact with municipal services and family to ensure appropriateness of social environment |
| Cognition | MMSE  Clock drawing test  Interview of relatives/caregivers | Further cognitive evaluation  Increased attention and planning of the postoperative phase |
| Psychological status | GDS  Patient/relative interview | Therapy  Drug change |
| Functional status | ADL (Katz index)  iADL  Battery of physical tests** | As mentioned under social status  Physical exercise program (mandatory)  Referral to occupational therapist |
| Nutrition | BMI  MNA  Weight loss (unintentional) within 3 months | Referral to dietitian  Protein supplement |
| Geriatric syndromes*** | Dementia  Delirium  Number of falls  Incontinence  Osteoporosis  Constipation |  |
|  | | |

**Supplement 2**

Exercise programme during hospitalisation: in case of laparotomy exercises are conferred with the surgeon. A trained physiotherapist carefully supervises all training sessions.

| Duration of exercise program | Depending on patient status. 10-30 minutes. |
| --- | --- |
| No. of repetitions (PRT) | 12-8 RM |
| No. of sets (PRT) | 3 sets of 10 |
| Resting period between sets (PRT) | 40-60 sec. (depending on patient) |
| No. of exercise sessions | Daily during stay at surgical ward. |
| Description of exercises | Bridge: Starting position: patient on the back (in bed) with knees bend and feet flat on the bed. Can be made with different levels of resistance depending of variation of starting position. Targets gluteal muscles, lower back and hamstrings.  Knee extensions: starting position: patient sitting in a bed or on a chair with sandbags strapped to the ankle. Resistance adjusted with weight of sandbags. Targets quadriceps muscles.  Calf-rasises: starting position: patient sitting on chair or standing on the floor depending of level of resistance. Targets gastrocnemius, tibialis posterior and the soleus muscle.  Sit to stand: starting position: patient sitting on a chair or elevated bed. Can be made with different levels of resistance depending on starting position and use/non-use of armrests. Targets Erector spinae, rectus femoris, vastus medialis, biceps femoris, gluteus maximus and rectus abdominus. |

**Supplement 3**

Endpoints

| Physical function tests | |
| --- | --- |
| **Endpoint** | **Details** |
| 30-second Chair stand test | Can be performed as a normal or modified version. The test will be carried out in accordance with the test manual (1). |
| Gait speed test (6 and 10 m) | Gait speed measures physical performance. Earlier studies have proved its predictive value for functional and cognitive decline, falls, hospitalisation, physical independence and mortality (2, 3). |
| Six-minute walk test | The six-minute walk test assesses aerobic capacity and endurance. It measures the distance covered over a time of 6 minutes walking. It has been tested in numerous diseases (e.g. cardiac, cancer and pulmonary diseases) (4, 5) and previous studies have demonstrated that the test has prognostic value among patients undergoing surgery for cancer (6). |
| Handgrip strength test | The handgrip strength test (HST) assessed with a Jamar dynamometer will be used to measure Upper-body strength. HST may be associated with QoL, functional capacity and survival in patients with cancer (7). |

**Supplement 4**

Postoperative complications (30 + 90 days) graded from 0-5 based on the classification system validated by Clavien and Dindo (8). Grade 0 represent no complications. Grade 1 any deviation from normal postoperative course. Grade 2 is any complication requiring pharmacological treatment including antibiotics. Blood transfusion and total parenteral nutrition are also included. Grade 3 are complications requiring surgical, radiological or endoscopic intervention. Grade 4 are life threatening complications requiring intermediate / intensive care unit management. Grade 5 is death.

**Supplement 5**

Serum biomarkers:

**Interleukin 6 (IL-6)** is an inflammatory cytokine elevated in serum of patients with CRC compared with healthy subjects. Several studies have investigated the potential use of IL-6 as a prognostic marker in patients with CRC but its value in older frail patients with CRC is not known (9). IL-6 will be measured with enzyme-linked immunosorbent assay (ELISA) from Quantikine HS600B, R&D Systems, Abingdon, UK.

**YKL-40,** also known as chitinase 3-like1 protein (CHI3L1), is an inflammatory protein mainly produced by cancer cells, macrophages, and neutrophils (10). Patients with cancer and high serum YKL-40 have shorter overall survival than patients with normal YKL-40 levels (11). YKL-40 will be measured with ELISA from Quidel, San Diego, CA, USA.

**Growth differentiation factor 11 (GDF11) and growth differentiation factor 11 (GDF15)** have previous been linked to disease and related to the loss of muscle as seen in cancer (12, 13).

GDF11 and GDF15 will be analysed with ELISA.

**Olink immuno-oncology protein panel** ([www.olink.com](http://www.olink.com)), a multiplex immune-assay using proximity extension assay. The panel have not been tested in older patients with colorectal cancer.

**Supplement 1 legend:**

Abbreviations: *CGA* Comprehensive Geriatric Assessment, *CIRS-G* Cumulative Illness Rating Scale-Geriatrics, *START* Screening Tool to Alert doctors to Right Treatment, *STOPP* Screening Tool of Older Person’s Prescriptions, *MMSE* Mini Mental State Examination, *GDS* Geriatric Depression Scale, *ADL* Activities of Daily Living, *iADL* instrumental Activities of Daily Living, *BMI* Body Mass Index, *MNA* Mini Nutritional Assessment.
*Biochemistry includes Vitamin D, TSH, folate, zinc, magnesium. If anaemia then folate, cobalamin, ferritin, transferrin receptor.
** Physical test includes 30-second chair stand test, 6+10 m gait speed, 6 min walking test, handgrip strength.

*** May overlap with other domains.

**Supplement 2 legend**

PRT Progressive resistance training, *RM* Repetition max

**References**

1. Rikli R JJ. Senior Fitness Test Manual-2nd Edition. Human Kinetics. 2001.

2. Guralnik JM, Ferrucci L, Pieper CF, Leveille SG, Markides KS, Ostir GV, et al. Lower extremity function and subsequent disability: consistency across studies, predictive models, and value of gait speed alone compared with the short physical performance battery. The journals of gerontology Series A, Biological sciences and medical sciences. 2000;55(4):M221-31.

3. Cesari M, Kritchevsky SB, Penninx BW, Nicklas BJ, Simonsick EM, Newman AB, et al. Prognostic value of usual gait speed in well-functioning older people--results from the Health, Aging and Body Composition Study. Journal of the American Geriatrics Society. 2005;53(10):1675-80.

4. Schmidt K, Vogt L, Thiel C, Jager E, Banzer W. Validity of the six-minute walk test in cancer patients. International journal of sports medicine. 2013;34(7):631-6.

5. Rikli RE JC. The Reliability and Validity of a 6-Minute Walk Test as a Measure of Physical Endurance in Older Adults. Journal of Aging and Physical Activity 1998;6:363-75.

6. Hayashi K, Yokoyama Y, Nakajima H, Nagino M, Inoue T, Nagaya M, et al. Preoperative 6-minute walk distance accurately predicts postoperative complications after operations for hepato-pancreato-biliary cancer. Surgery. 2017;161(2):525-32.

7. Kilgour RD, Vigano A, Trutschnigg B, Lucar E, Borod M, Morais JA. Handgrip strength predicts survival and is associated with markers of clinical and functional outcomes in advanced cancer patients. Supportive care in cancer : official journal of the Multinational Association of Supportive Care in Cancer. 2013;21(12):3261-70.

8. Dindo D, Demartines N, Clavien PA. Classification of surgical complications: a new proposal with evaluation in a cohort of 6336 patients and results of a survey. Annals of surgery. 2004;240(2):205-13.

9. Vainer N, Dehlendorff C, Johansen JS. Systematic literature review of IL-6 as a biomarker or treatment target in patients with gastric, bile duct, pancreatic and colorectal cancer. Oncotarget. 2018;9(51):29820-41.

10. Libreros S, Iragavarapu-Charyulu V. YKL-40/CHI3L1 drives inflammation on the road of tumor progression. J Leukoc Biol. 2015;98(6):931-6.

11. Bian B, Li L, Yang J, Liu Y, Xie G, Zheng Y, et al. Prognostic value of YKL-40 in solid tumors: a meta-analysis of 41 cohort studies. Cancer cell international. 2019;19:259.

12. Glass DJ. Elevated GDF11 Is a Risk Factor for Age-Related Frailty and Disease in Humans. Cell metabolism. 2016;24(1):7-8.

13. Cardoso AL, Fernandes A, Aguilar-Pimentel JA, de Angelis MH, Guedes JR, Brito MA, et al. Towards frailty biomarkers: Candidates from genes and pathways regulated in aging and age-related diseases. Ageing research reviews. 2018;47:214-77.
